# Immuno-modulatory effect of local rhEGF treatment during tissue repair in diabetic ulcers

**DOI:** 10.1530/EC-18-0117

**Published:** 2018-03-28

**Authors:** Natalio García-Honduvilla, Alberto Cifuentes, Miguel A Ortega, Marta Pastor, Garazi Gainza, Eusebio Gainza, Julia Buján, Melchor Álvarez-Mon

**Affiliations:** 1 Department of Medicine and Medical SpecialitiesFaculty of Medicine and Health Sciences, University of Alcalá, Alcalá de Henares, Madrid, Spain; 2 Networking Biomedical Research Center on BioengineeringBiomaterials and Nanomedicine (CIBER-BBN), Madrid, Spain; 3 Ramón y Cajal Institute of Sanitary Research (IRYCIS)Madrid, Spain; 4 University Center of Defense of Madrid (CUD-ACD)Madrid, Spain; 5 Biopraxis Research AIEÁlava, Spain; 6 Immune System Diseases-Rheumatology and Oncology ServiceUniversity Hospital Príncipe de Asturias, Alcalá de Henares, Madrid, Spain

**Keywords:** wound healing, diabetes mellitus, diabetic foot ulcer, rhEGF

## Abstract

Wound healing is a complex process that can be severely impaired due to pathological situations such as diabetes mellitus. Diabetic foot ulcers are a common complication of this pathology and are characterized by an excessive inflammatory response. In this work, the effects of local treatment with recombinant human epidermal growth factor (rhEGF) were studied using a full-thickness wound healing model in streptozotocin-induced diabetic rats. Wound healing process was assessed with different concentrations of rhEGF (0.1, 0.5, 2.0 and 8.0 µg/mL), placebo and both diabetic and non-diabetic controls (*n* = 53). The macroscopic healing observed in treated diabetic rats was affected by rhEGF concentration. Histologically, we also observed an improvement in the epithelialization, granulation tissue formation and maturation in treated groups, finding again the best response at doses of 0.5 and 2.0 µg/mL. Afterwards, the tissue immune response over time was assessed in diabetic rats using the most effective concentrations of rhEGF (0.5 and 2.0 µg/mL), compared to controls. The presence of macrophages, CD4^+^ T lymphocytes and CD8^+^ T lymphocytes, in the reparative tissue was quantified, and cytokine expression was measured by quantitative real-time PCR. rhEGF treatment caused a reduction in the number of infiltrating macrophages in the healing tissue of diabetic, as well as diminished activation of these leukocytes. These findings show that local administration of rhEGF improves the healing process of excisional wounds and the quality of the neoformed tissue in a dose-dependent manner. Besides, this treatment reduces the local inflammation associated with diabetic healing, indicating immuno-modulatory properties.

## Introduction

Wound healing is a dynamic process, involving different components of the extracellular matrix (ECM), soluble mediators, several resident cell lines and infiltrating leukocytes. Cutaneous wounds trigger cellular and tissue responses, including clotting, platelet activation, inflammatory cell infiltration, re-epithelialization, granulation tissue formation, ECM deposition, wound contraction and tissue maturation (1).

The wound healing process can become impaired due to pathological situations, as in patients suffering from diabetes and/or chronic ulcers of different aetiologies (2). Diabetes mellitus is a systemic disease characterized by an abnormal carbohydrate metabolism with chronic hyperglycaemia and secondary involvement of different organs including vascular and peripheral nervous system.

According to the World Health Organization, in the year 2000, at least 171 million people suffered from diabetes, and it is estimated that 285 million people (aged 20–79 years) suffered from this disease in 2010 (3, 4). In the United States alone, more than 200,000 deaths occur each year among patients with diabetes. Diabetes was the seventh leading cause of death in 2013 and 29 million people (9.3% of the US population) are estimated to have diagnosed or undiagnosed diabetes (5). The lifetime risk of a foot ulcer in patients with diabetes may be as high as 25 percent (6). Diabetic foot ulcers are a major cause of morbidity and mortality, accounting for approximately two-thirds of all nontraumatic amputations performed in the United States (7).

Multiple factors contribute to the formation of diabetic foot ulcers, such as oxidative stress, altered inflammatory response or cutaneous microcirculation deficiencies. The pathogenesis of these wounds is complex, and different mechanisms are involved; peripheral neuropathy, mechanical pressure due to structural deformities of the foot, macro- and microvascular impairments and infection contribute to the chronicity of this kind of ulcers (8). Inadequate secretion of ECM proteins (9), dysregulation of macrophage activity (10, 11), proliferation and increased apoptosis of cutaneous fibroblasts, impaired angiogenesis, and re-epithelialization (12, 13) have all been described in the literature. In order to model this kind of wounds, the experimental induction of diabetes mellitus with streptozotocin in rodents has been widely used, which causes an impaired wound healing response in the diabetic animals (14, 15).

Due to the clinical relevance of diabetic foot ulcers and their difficult treatment, new therapeutic alternatives are under investigation. Compressive therapies, advanced dressings, cell therapy and the use of bioactive molecules to modulate the wound healing process are among the most widely used treatments (10, 16). New therapies for diabetic foot ulcer include the modulation of the cellular and molecular mechanisms involved in wound healing. In relation with this, it is known that re-epithelialization and granulation tissue formation are primarily regulated by the release of diverse endogenous growth factors, such as the epidermal growth factor (EGF), the fibroblast growth factors family, the transforming growth factor alpha (TGF-α) and the platelet-derived growth factor (PDGF) (1, 17). Currently, the therapeutic use of local administration of growth factors such as EGF is under investigation in diabetic foot ulcers (18). Besides, intralesional administration of recombinant human EGF (rhEGF) has been proven to enhance granulation tissue formation in patients suffering from Wagner’s Grade 2 and 3 ulcers with elevated risk of amputation, achieving total closure between weeks 19 and 27 and avoiding limb loss in more than 65% of patients after a one-year follow-up (19). The administration of rhEGF appears to promote the proliferation and differentiation of mesenchymal and epithelial cells. However, the patterns of cellular growth and tissue repair induced by the local administration of different doses of rhEGF have not been established. Furthermore, the potential effects of the intralesional rhEGF administration in the local inflammatory-immune response remain unknown (19, 20, 21).

The aim of the present work was to study of the effects of the local administration of rhEGF upon the wound healing process, in a full-thickness excisional cutaneous defect, using an experimental model of diabetes induced by streptozotocin. We analysed the quality of the regenerative tissue and the patterns of inflammatory-immune cells infiltration induced by the administration of different doses of rhEGF.

## Materials and methods

### Experimental design

Female Wistar rats (*n* = 101), weighing between 185 and 247 g (mean 222.00 ± 11.85 g), were used in this study. All procedures were carried out according to the recommendations detailed in the Guide for the Care and Use of Laboratory Animals of the National and European Institutes of Health (Spanish law 32/2007, Spanish Royal Decree 53/2013, European Directive 2010/63/UE and European Convention of the Council of Europe ETS123).

Two different experimental protocols were carried out to study the wound healing process in diabetic rats (DRs) and healthy control rats (HRs) and the effect upon it of the local treatment with rhEGF. Protocol 1 (*n* = 53) was used for the macroscopic and microscopic analyses of the reparative process and the evaluation of different doses. After this, Protocol 2 (*n* = 48) was performed to study the immune response during wound healing using the doses previously selected. The experimental groups in both protocols are detailed in Table 1.

**Table 1:** Experimental groups in protocol 1 and protocol 2 experimental designs.

Group		Diabetes	Treatment	Time of study (days)
Protocol 1				
HR	(*n* = 6)	No	None	21
DR-CTRL	(*n* = 6)	Yes	None	21
DR-PLCB	(*n* = 6)	Yes	Vehicle (0.9% NaCl)	21
DR-0.1	(*n* = 6)	Yes	0.1 µg/mL rhEGF	21
DR-0.5	(*n* = 6)	Yes	0.5 µg/mL rhEGF	21
DR-2.0	(*n* = 6)	Yes	2.0 µg/mL rhEGF	21
DR-8.0	(*n* = 6)	Yes	8.0 µg/mL rhEGF	21
Protocol 2				
HR	(*n* = 12)	No	None	3, 7, 14, 21
DR-PLCB	(*n* = 12)	Yes	Vehicle (0.9% NaCl)	3, 7, 14, 21
DR-0.5	(*n* = 12)	Yes	0.5 µg/mL rhEGF	3, 7, 14, 21
DR-2.0	(*n* = 12)	Yes	2.0 µg/mL rhEGF	3, 7, 14, 21

### Induction of diabetes mellitus

Experimental diabetes was induced by intraperitoneal injection of streptozotocin (Sigma-Aldrich) at a dose of 70 mg/kg in citrate buffer (5 mM, pH 4.5), followed by a second administration after 24 h. The increase of glycosuria was monitored using reactive strips, and experimental diabetes was considered stable after four weeks. Additionally, histological examination of the pancreas was carried out at the end of the study, showing alterations in the morphology and distribution of Langerhans islets in DRs.

### Cutaneous wounding

All animals were anaesthetized by intraperitoneal administration of 150 mg/kg ketamine (Imalgene 1000, Merial-Sanofi, Barcelona, Spain) and 2 mg/kg xylazine (Rompún, Bayer, Leverkusen, Germany). Following this, an excisional circular defect (diameter = 1.5 cm) was created, involving all cutaneous and subcutaneous tissues. These lesions were situated on the proximal back region of the animals, in order to avoid manipulation. The wounds were observed and cleaned three times per week, until the end of the study.

### Local treatment of the skin defects

**Figure 1:**
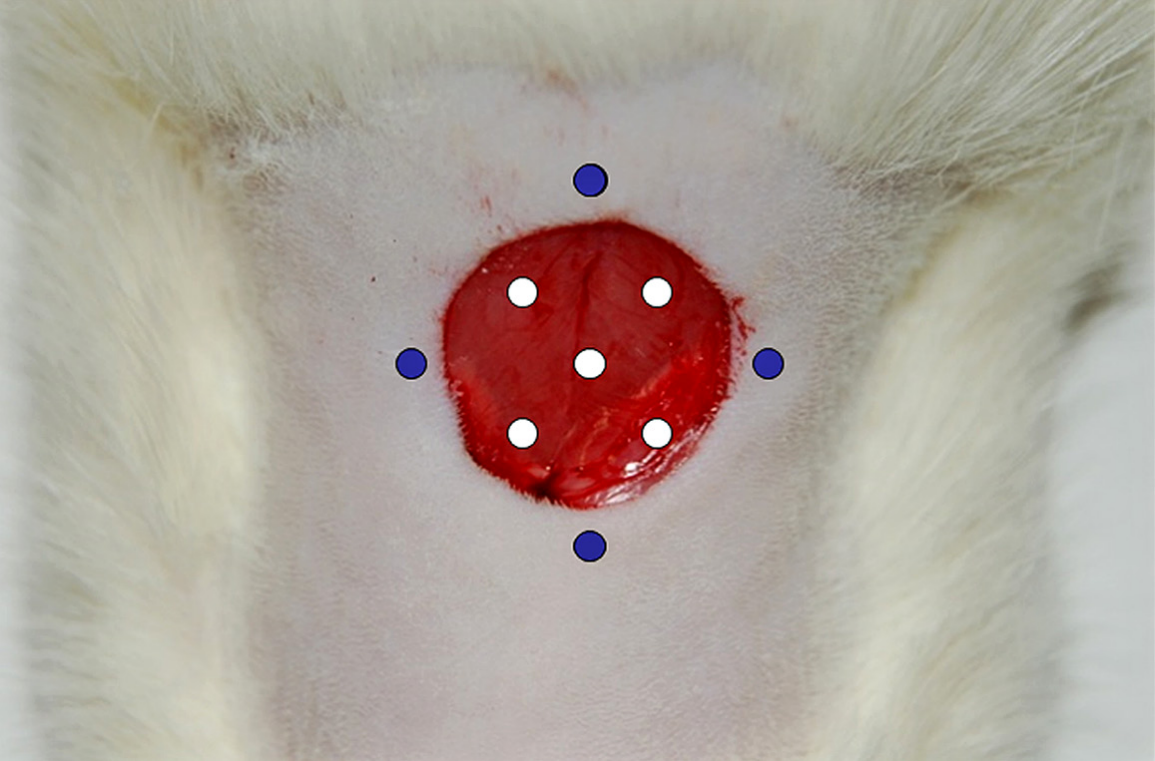
Injection pattern followed in the administration of rhEGF. Perilesional administration (blue spots): 4 subcutaneous injections, 1.1 mL/point. Intralesional administration (white spots): 5 intramuscular injections, 1.1 mL/point.

Both placebo (physiological saline) and rhEGF (Nepidermina, Praxis Pharmaceutical S.A. Madrid, Spain) at determined doses were administered by subcutaneous perilesional injection in four points and by intralesional injection in five points, as shown in Fig. 1. Each point received a volume of 0.11 mL, for a total volume of 1 mL per administration and animal.

Placebo and rhEGF treatments were dispensed 6 times in 14 days (3 doses/week in alternate days), starting the day of the surgical wounding (*t* = 0 day).

### Morphological and morphometrical studies

The evolution of the wound area of animals from Protocol 1 was assessed three times per week, until the day the animals were killed (*t* = 21 days), and photographs of each animal were taken. The re-epithelialized area of the wound was measured using ImageJ software (NIH).

At day 21, wound tissue samples were excised, fixed in 10% buffered formaldehyde and embedded in paraffin for histological and immunohistochemical studies. Serial sections of the tissue (5–8 µm) were submitted to basic histological techniques (haematoxylin–eosin and Masson’s trichrome stainings). The presence of collagen in the neoformed tissue, as well as its maturation and organization degrees, was assessed using Sirius Red differential staining.

Samples were evaluated in a Zeiss AxioPhot microscope equipped with a digital camera AxioCam HRc (Carl Zeiss), and morphometric studies were performed using ImageJ software. The thickness of the dermal and epidermal layers was measured in both repaired and undamaged zones, paying special attention to the degree of organization and compaction of neodermis and neo-epidermis.

### Immunohistochemical studies

The presence of macrophages, CD4^+^ T lymphocytes and CD8^+^ T lymphocytes, was assessed immunohistochemically using the avidin–biotin complex technique in animals from Protocol 2.

Macrophages were detected using a mouse anti-CD68 monoclonal antibody (AbD Serotec, Kidlington, UK), and CD4^+^ and CD8^+^ lymphocytes were immunolabelled using mouse anti-CD4 and anti-CD8 monoclonal antibodies (Labgen/NatuTec, Frankfurt, Germany). After deparaffinization, slides were blocked with 3% BSA, incubated with primary antibody (CD68: 1/100; CD4, CD8: 1/50), followed by biotinylated goat anti-mouse IgG antibody and avidin–alkaline phosphatase conjugate (1/300 and 1/200 respectively; Sigma-Aldrich).

Slides were developed using Fast Red (Sigma-Aldrich) chromogenic substrate and nuclei were contrasted with haematoxylin. Negative controls exposed to blocking solution instead of primary antibodies were included in all samples. For each technique, labelled cells were quantified in 10 microscopic fields (40× magnification) per sample, and results are expressed as mean number of positive cells/field.

### Real-time PCR

The expression of several key cytokines in the wound tissue was measured by quantitative real-time PCR. Total RNA was isolated from skin frozen samples of animals from Protocol 2 using TRIzol (Thermo Fisher Scientific), and its quantity and purity were assessed in a NanoDrop ND-1000 spectrophotometer (Thermo Fisher Scientific). A total of 200 ng of total RNA was reverse-transcribed with oligo-dT primers and the M-MLV reverse transcriptase enzyme (Thermo Fisher Scientific). cDNA was quantified by real-time PCR, using the relative standard curve method, in a StepOnePlus System (Thermo Fisher Scientific). Primer sequences and conditions are described in Table 2, and thermal cycling conditions were as follows: initial stage of 10 min at 95°C, followed by 45 cycles of 15 s at 95°C, 30 s at different temperatures (Table 2) and 1 min at 72°C. Negative controls were run in each reaction, and PCR products were submitted to 2% agarose gel electrophoresis. Gene expression was normalized using GAPDH as reference gene.

**Table 2:** Primer sequences, annealing temperatures and amplicon sizes of the studied genes in real-time PCR experiments.

Gene	Sequence (5′→3′)		Temp. (°C)	Amp. size (bp)
GAPDH	FwdRev	TGA ACG GGA AGC TCA CTG GTCC ACC ACC CTG TTG CTG TA	60	306
IL-2	FwdRev	CTG ACG CTT GTC CTC CTT GTCTGC TGC TGT GTT TCC TTT GC	60	64
IL-10	FwdRev	CAT GGC CCA GAA ATC AAG GAAGC GTC GCA GCT GTA TCC A	60	75
IL-12p40	FwdRev	TCA CCT GGA CCT CAG ACC AGAGAA CCG TCC GGA GTA GTT TGG	63	239
TNF-α	FwdRev	CTG TCT ACT GAA CTT CGG GGT GGAG GCT GAG TTT CTC CTG GTA TG	60	370

### Statistical analysis

GraphPad Prism 5 software (GraphPad Software) was used for data analysis. ANOVA followed by Tukey–Kramer test was used to compare data between groups. Data are expressed as mean ± s.e.m., and levels of significance were set at *P* ≤ 0.05 (*); *P* ≤ 0.01 (**); *P* ≤ 0.001 (***).

## Results

### Morbidity and mortality

Of 53 animals included in Protocol 1, 11 did not complete the experimental phase due to the aggressiveness of the diabetic process, the anaesthetic procedures and/or the presence of seroma, with the mobility–mortality rate being 20.75%. All 48 animals from Protocol 2 reached the end of the study.

During the study, the group of healthy rats increased their body weight by 5.71% (initial mean weight: 221.75 ± 6.95 g; final mean weight: 234.42 ± 18.20 g). In contrast, DRs suffered a loss of weight of 17.54% (initial mean weight: 222.46 ± 6.69 g; final mean weight: 183.44 ± 10.49 g).

In each animal, glycosuria was monitored using reactive strips (Carulla Vekar, HISPAN LAB SA, Madrid, Spain) every 48 h until stable maximal glycosuria was reached. Thus, experimental diabetes was considered stable after four weeks, and glycosuria was monitored weekly. The observed hyperglycaemia-related polyuria may be a cause for the described weight loss on diabetic animals.

### Topical treatment with rhEGF normalizes the macroscopic wound healing in diabetic rats

During the first two weeks of the study, period when the rhEGF treatment was administered three times per week, the reparative process progressed without achieving complete re-epithelialization in any group.

**Figure 2:**
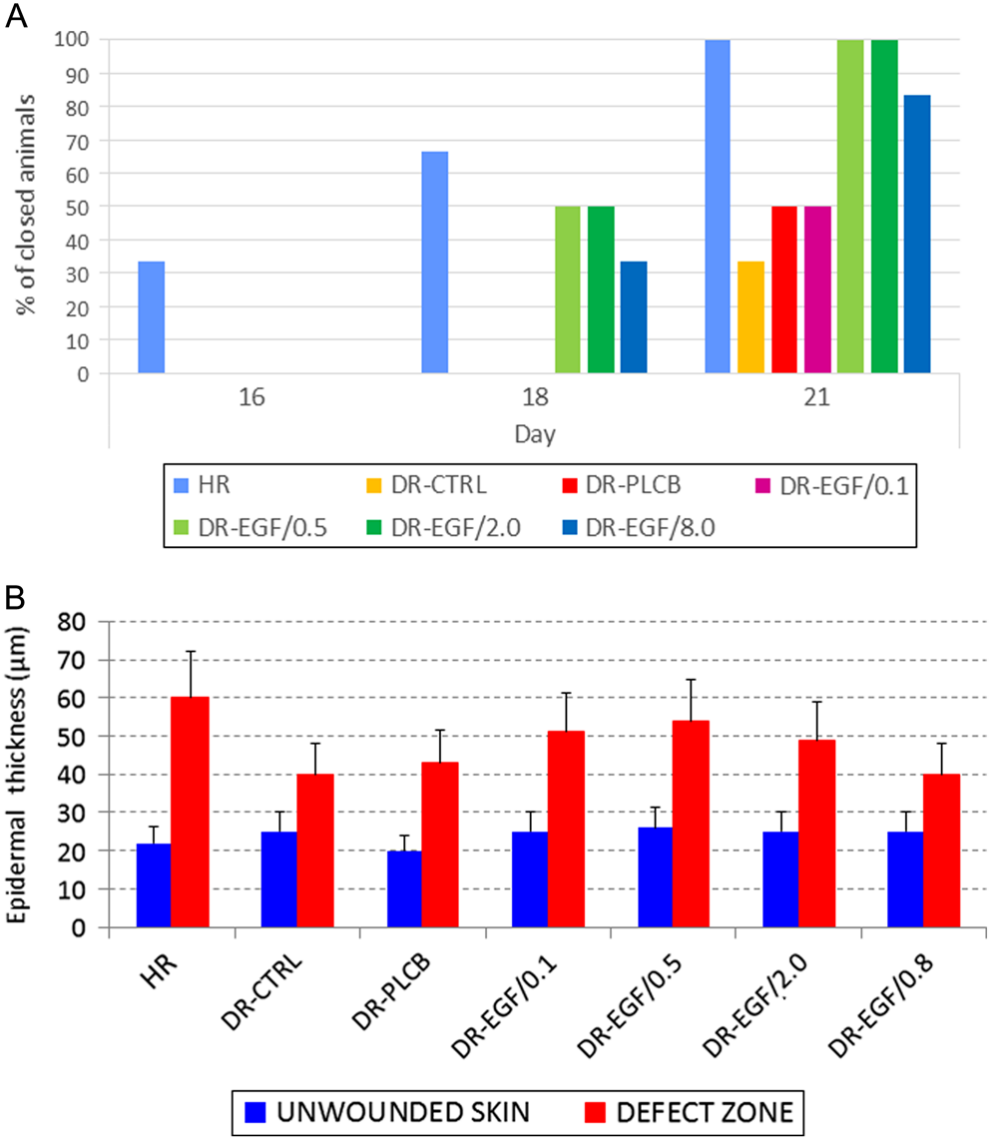
(A) Complete closure of the defect. Results are expressed as cumulative percentage of animals presenting a closed wound at days 16, 18 and 21 after surgery. (B) Differences in the epidermal thickness between unwounded skin and healed tissue in the different study groups. Results are expressed as mean ± s.d. (µm).

The evolution of the healing in HR, used as reference of normality, showed at 48 h the presence of a peripheral granulation tissue generating from the healthy edges, followed by a progressive centripetal epithelialization of the defect area. At day 14, this process was still in progress, while at day 21, all animals had reached solution of continuity (Fig. 2).

In DRs without rhEGF treatment (DR-CTRL and DR-PLCB), the cicatricial process evolved in a significantly slower fashion than in healthy group. In addition, at day 21, only 1 out of the 6 DR-CTRL animals had achieved a complete closure of the defect (Fig. 2).

Animals treated with 0.1 µg/mL rhEGF showed a macroscopic evolution of the wound similar to the group of untreated DRs, and slower than healthy controls. At day 21, none of the 6 animals reached complete closure of the wound. In contrast, treatment with either 0.5 or 2.0 µg/mL rhEGF induced a reparative process similar to that observed in non-diabetic rats. However, in rats treated with 8.0 µg/mL rhEGF, a significantly slower healing process was observed, compared to healthy controls. At day 21, 5 out of the 6 (83.33%) animals from this group had achieved a complete closure (Fig. 2).

### Different doses of local administered rhEGF induce different patterns of histological wound healing in diabetic rats and an optimal dose is found

Histological specimens from Protocol 1 animals were evaluated microscopically to assess the progress and quality of the reparative process. In all the experimental groups, the measurement of the epidermal thickness showed higher values in the repaired area, compared to normal skin of the unwounded borders. Neo-epidermal thickness in HR group was found to be significantly greater than in the rest of the groups, while differences were also observed among diabetic groups. DR-EGF/0.1, DR-EGF/0.5 and DR-EGF/2.0 groups showed a thicker neo-epidermis than DR-CTRL, DR-PLCB and DR-EGF/8.0 groups (Fig. 2).

**Figure 3:**
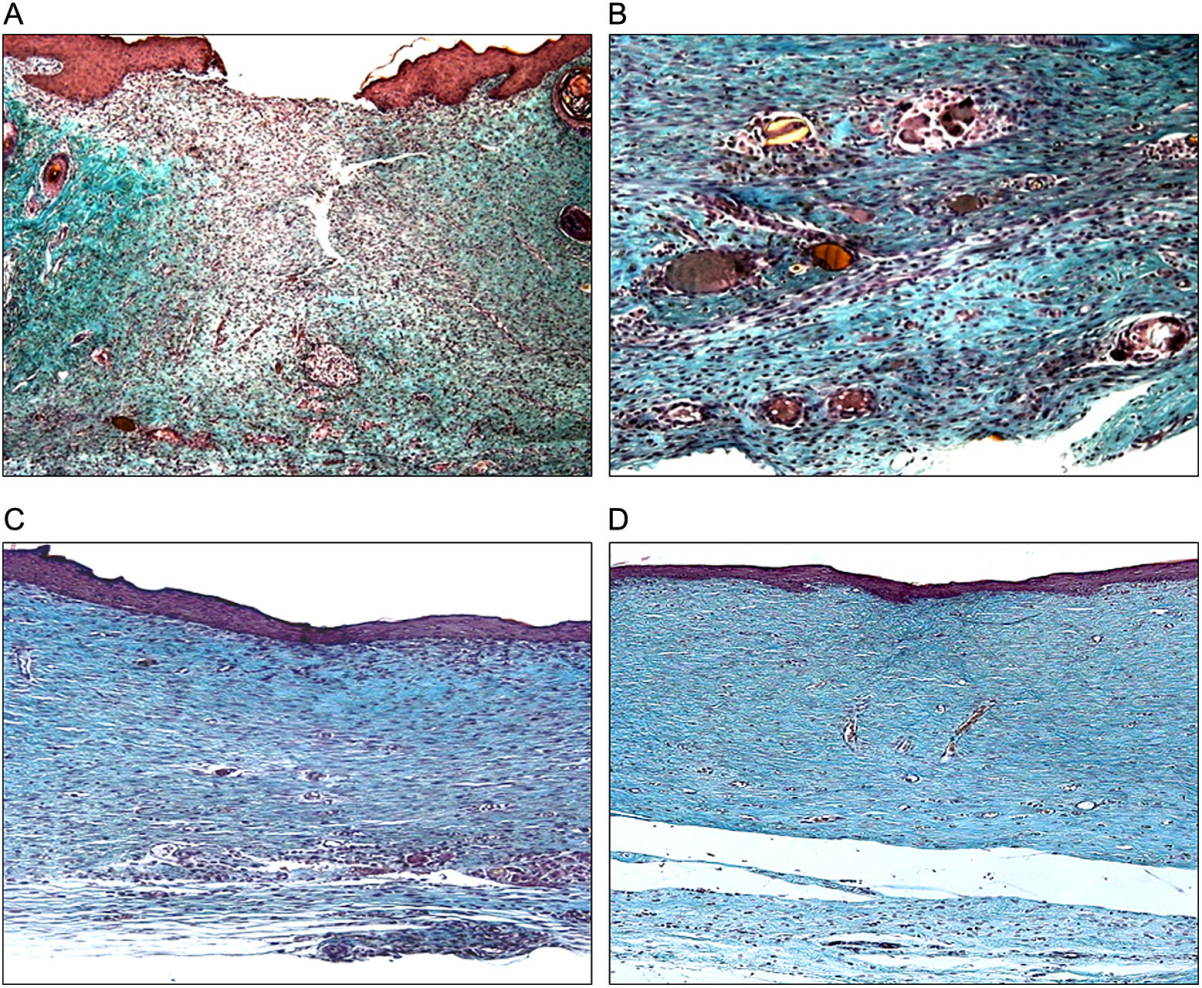
Histological examination of the scar tissue at day 21 with Masson’s trichrome staining. (A) DR-CTRL group, aspect of healing area of one of the animals that did not complete wound closure (OM 5×). (B) DR-CTRL group. Foreign body and inflammatory infiltration in the deep tissue of a fully closed animal (OM 20×). (C) DR-EGF/0.5 group. Epidermis of the repaired tissue (OM 10×). (D) DR-EGF/2.0 group. Dermal–epidermal junction organization (OM 5×). Bar = 200 µm.

HR animals showed a neoformed cutaneous tissue constituted by the usual layers (epidermal, dermal and subcutaneous), with an organization similar to the native tissue. The area of the defect was characterized by a thicker epidermis, as described earlier, and an underlying dense connective tissue. This neodermis showed a high degree of contraction, while skin appendages were absent in the repaired tissue. In DR-CTRL group, we could observe a loose neotissue, and the reparative epidermis showed characteristics of a non-consolidated epithelium, with an irregular dermo-epidermal transition. Compared to the native dermis, the new tissue presented a reduced density, both in cell number and in collagen presence and organization (Fig. 3). DR-PLCB animals showed a similar behaviour as that observed in diabetic control rats. All animals from both groups, regardless of having reached a complete closure or not, presented a chronic inflammatory infiltrate in the whole area of the lesion. Foreign body reaction could be observed in the deep dermal zone, accompanied by macrophages and plasmacyte accumulation, while the undamaged zone presented a moderate mast cell population and some eosinophils.

In rats from DR-EGF/0.1, the repaired tissue was constituted by a dense connective tissue lacking dermal papillae. Collagen appeared uniformly organized in most zones of the repaired skin. In the deep neodermis, collagen fibre packing and maturation was greater, and the bundles showed a higher degree of compaction. The transition zone to the unwounded skin was evident due to the absence of hair follicles and the different organization of the mature dermal ECM. DR-EGF/0.5 and DR-EGF/2.0 groups showed similar histologic patterns including the vascular network in the reticular dermis. However, this vascular network did not reach this level of development in DR-EGF/0.1 group (data not shown) (Fig. 3). Collagen maturation in the inner two-thirds of the reticular neodermis was observed in both groups, while in the papillary dermis of DR-EGF/0.5 animals, the collagen was thinly distributed, in contrast to DR-EGF/2.0 rats, which presented a more uniform collagenization. In DR-EGF/8.0 group, repaired skin was populated by numerous highly differentiated fibrocytes, surrounded by dense and organized collagen bundles and blood vessels in the papillary dermis. The medial zone was moderately less dense, while the deep zone was again highly vascularized. This group presented a high tissue reactivity, affecting not only the repaired tissue, but also the deep adipose tissue, which showed signs of activation, with disorganized cell proliferation, abundant angiogenesis and marked increase of fibrosis.

The observation of the patterns of collagen presence and maturation in the repaired area indicated a higher degree of maturation in DRs treated with 0.5 and 2.0 µg/mL rhEGF compared to DR-EGF/0.1 and DR-EGF/8.0 animals, but inferior to that observed in healthy animals.

### rhEGF treatment diminishes infiltration and activation of macrophages in the healing tissue of diabetic rats

**Figure 4:**
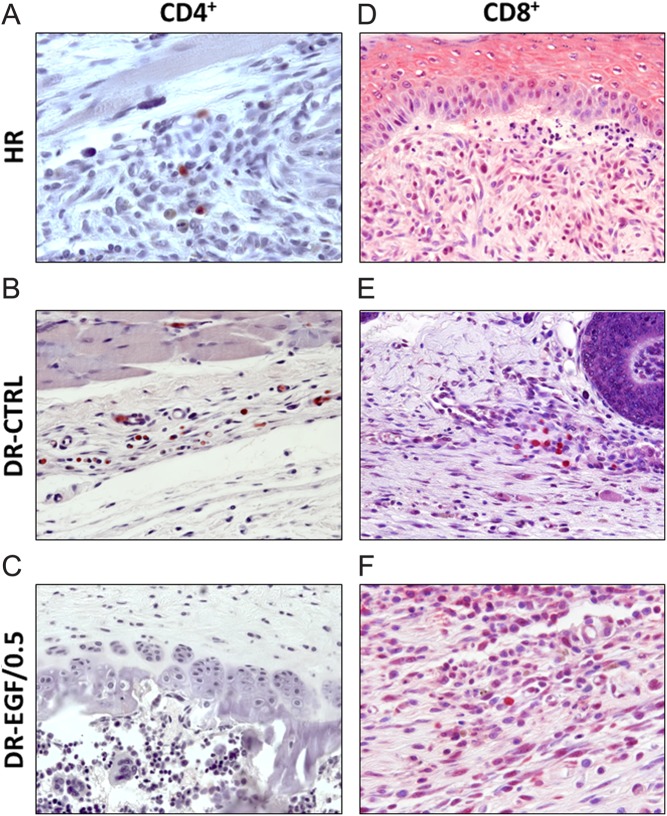
Presence of T lymphocytes (CD4^+^ and CD8^+^) in the different groups at day 21: Minimal infiltration was observed in all groups, both for CD4^+^ (left panel) and CD8^+^ (right panel). (A) Presence of CD4^+^ T cells in HR group (OM 64×); (B) presence of CD4^+^ T lymphocytes in DR-PLCB group (OM 40×); (C) presence of CD4^+^ T lymphocytes in DR-EGF/0.5 group (OM 40×); (D) presence of CD8^+^ T lymphocytes in HR group (OM 40×); (E) presence of CD8^+^ T cells in DR-PLCB group (OM 40×); (F) presence of CD8^+^ T lymphocytes in DR-EGF/0.5 group (OM 64×). Bar = 50 µm.

**Figure 5:**
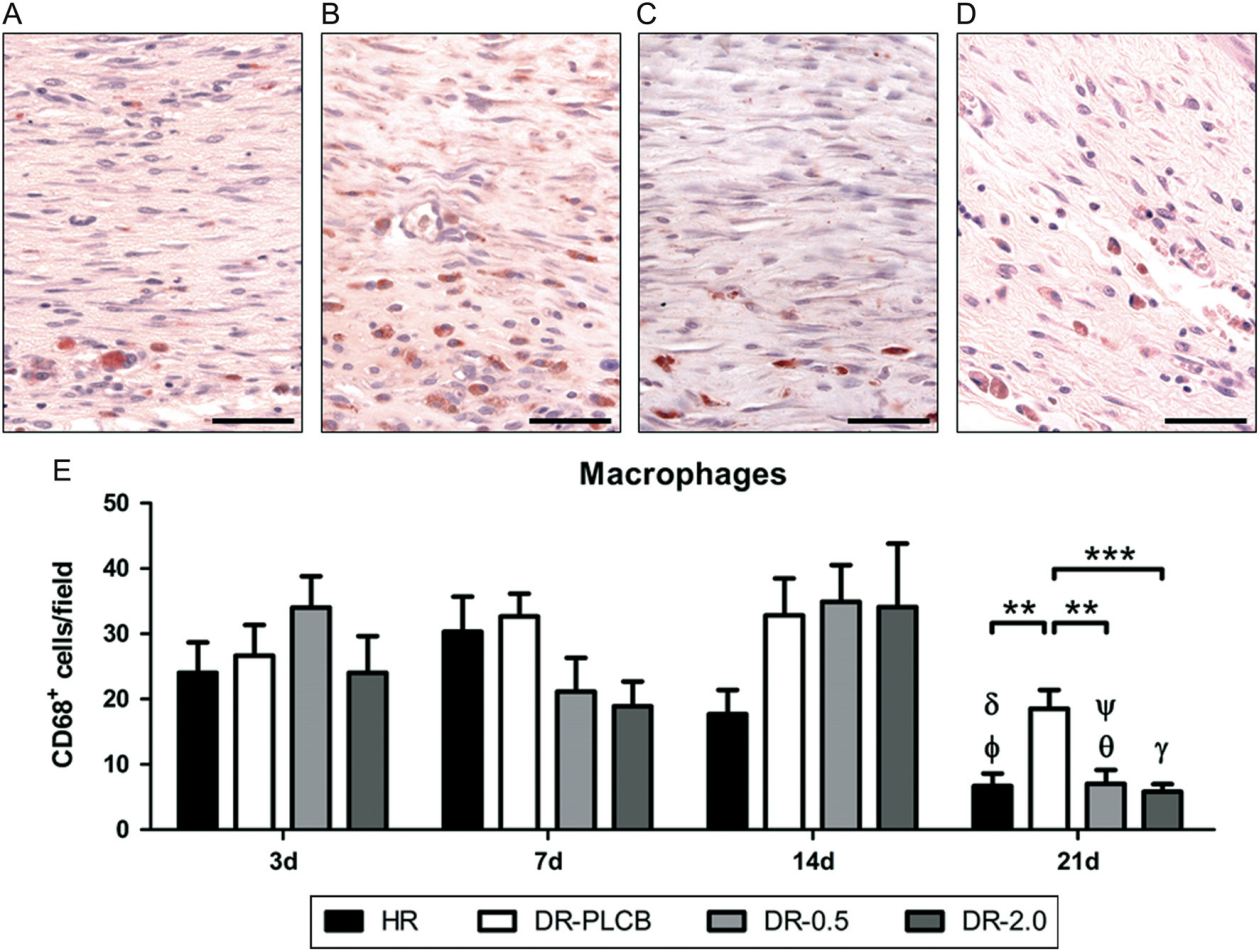
Presence of macrophages in the neo-dermal area: (A, B, C and D) CD68^+^ cells at day 21. Bar = 50 µm. (A) HR group (OM 40×); (B) DR-PLCB group (OM 40×); (C) DR-EGF/0.5 group (OM 40×); (D) DR-EGF/2.0 group (OM 40×); (E) evolution of the wound macrophage presence over time (3–21 day), expressed as number of CD68^+^ cells/field. (δ): * vs HR 3 days; (ϕ): ** vs HR 7 days; (ψ): ** vs DR-0.5 3 dyas; (θ): *** vs DR-0.5 14 days; (γ): ** vs DR-2.0 14 days.

After assessing the pro-reparative effects of rhEGF treatment in the wound healing process of diabetic wounds, the most effective concentrations of the growth factor (0.5 and 2.0 µg/mL) were routinely used in Protocol 2 to evaluate the effect of local rhEGF administration on the tissue immune response over time. Both healthy and DRs showed a minimal infiltration of CD4^+^ T lymphocytes and CD8^+^ T lymphocytes (Fig. 4). The sparse CD4^+^ T lymphocytes detected used to be localized in perivascular zones, while no specific pattern of presence was observed for the small amount of CD8^+^ lymphocytes. There were no significant quantitative differences in CD4^+^ and CD8^+^ T lymphocyte wound infiltration between the different groups of rats (data not shown). In contrast to the T lymphocyte response, a marked presence of monocytes/macrophages in the wound was already found in HR at day 3. This cellular infiltration peaked at day 7, progressively reducing their numbers at day 14 and significantly at day 21. DR-PLCB presented a similar behaviour at days 3 and 7, but showed a greater persistence of monocytes/macrophages at days 14 and 21, compared to control animals. Groups treated with rhEGF followed a similar pattern until day 14, but achieved a significant reduction in the number of tissue monocytes/macrophages at day 21 compared to DR-PLCB group. At this time point, both DR-EGF/0.5 and DR-EGF/2.0 groups showed a monocyte/macrophage presence in the neoformed tissue comparable to that of healthy rats (Fig. 5).

**Figure 6:**
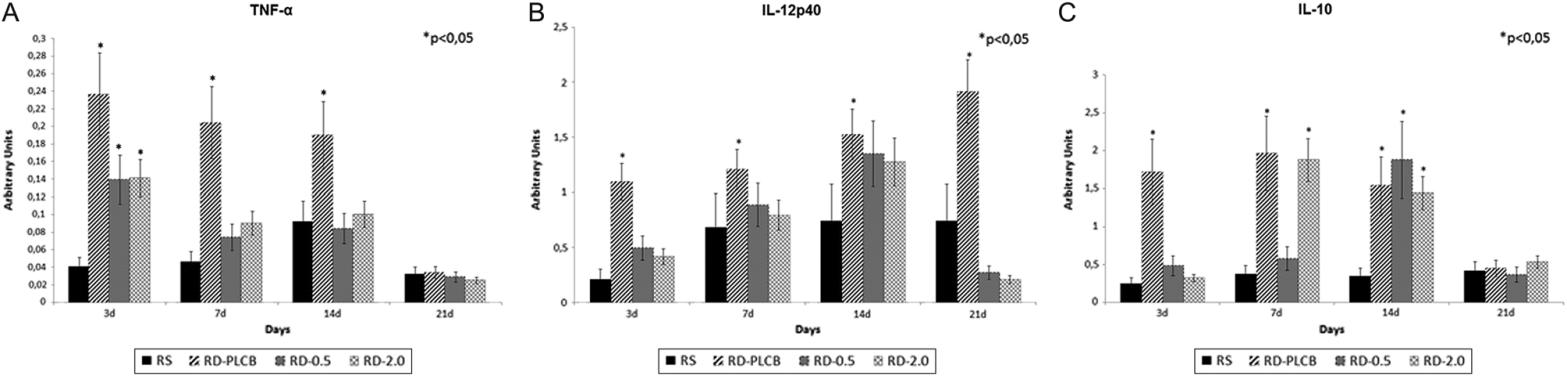
Evolution of the cytokine gene expression over time (3–21 days), measured by qRT-PCR. Results are expressed as arbitrary units. (A) TNF-α; (B) IL-12p40; (C) IL-10.

Gene expression of the cytokines TNF-α, IL-2, IL-10 and IL-12p40 in the healing tissue was measured using quantitative real-time PCR. Three days after wounding, TNF-α mRNA levels were higher in DRs compared to healthy controls, with DR-PLCB group showing the highest values. The expression of this cytokine started to decline at day 7 in diabetic groups, and equated to HR at day 21 (Fig. 6).

IL-12p40 mRNA expression in HR showed a slight increase over time, but without significant differences. At day 3, the expression of this cytokine was upregulated in diabetic animals, and in DR-PLCB group, mRNA levels kept rising until day 21. In contrast, RD-EGF/0.5 and RD-EGF/2.0 experience a decrease in IL-12p40 levels at day 21, with levels similar to HR and significantly lower than placebo group (Fig. 6).

No significant changes were observed in the expression of IL-10 in healthy animals. However, mRNA levels of this cytokine in DR-PLCB animals suffered an increase at day 3, which normalized progressively until day 21. DR-EGF/0.5 and DR-EGF/2.0 showed levels at day 3 similar to HR, followed by an increase at days 7 and 14, to finally normalize at the end of the study (Fig. 6).

Finally, the measurement of IL-2 mRNA showed levels below the dynamic range of the assay, indicating a low level of expression for this cytokine in all groups.

## Discussion

In this work, we have demonstrated that local treatment with rhEGF of excisional lesions in an experimental model of diabetes mellitus can improve the wound healing process and the quality of the neoformed tissue. This tissue repair effect of rhEGF is associated to a reduction of the local infiltration by inflammatory-immune cells. The pattern of effects of locally administrated rhEGF in the diabetic wound healing varies between different doses, but an optimal dose is defined.

Wound healing is a complex and well-orchestrated phenomenon, involving multiple cell lineages, including epithelial, mesenchymal, inflammatory and haematopoietic cells (22, 23). To achieve a good-quality healing, repair must be accompanied by an organized set of mechanisms and reconstruction of the cellular and ECM components of the injured tissue, starting with neutrophil infiltration and a second wave of macrophages, followed by granulation tissue formation and angiogenesis, fibroblast and myofibroblast colonization, contraction, maturation and re-epithelialization of the repaired tissue (24, 25, 26). Different local and systemic mechanisms can alter this process, leading to impaired wound healing. Diabetes mellitus is a metabolic disorder that severely affects wound repair, and can lead to well-known clinical problems like diabetic foot ulcers (7, 27). Our findings clearly show that diabetes causes a slowing of the reparative process, which even has led in some of the diabetic animals to a failure of the wound closure within our time of study. This impairment was also evident in the quality of the diabetic cicatricial tissue, which presented clear deficiencies relative to that of healthy controls, such as a reduced deposition of collagen and an altered epithelialization. These results agree with the established concept of altered wound healing in this diabetic model.

These alterations of the cutaneous repair in patients suffering from diabetes mellitus have prompted extensive research of potential treatments that could allow a better healing. Among the different therapeutic approaches that have been tested, the topical administration of EGF has been proposed as a promising agent (18, 19, 20, 28, 29), showing efficacy in the treatment of diabetic foot ulcers (30). EGF molecular family has been related to the regulation of cutaneous wound repair, and these molecules and their receptors are present in the tissues and cells involved in this process, such as keratinocytes, fibroblasts and mesenchymal cells (17, 31).

In our experimental model, we have ascertained that peri/intralesional administration of rhEGF induced an acceleration of the wound healing in DRs, making it able to equal the healthy response at optimal doses. This effect was accompanied by improvements at the histological level, promoting a better epithelialization and granulation tissue formation. Diabetic animals possessed a neo-epidermal layer that was thinner than in HR. However, local treatment with 0.5 and 2.0 µg/mL rhEGF showed a tendency to normalize this parameter at day 21. This effect might be related to the activity of this growth factor upon macrophage activation, which favours an increased angiogenesis, proliferation and migration of keratinocytes and a greater fibroblastic function (32, 33), leading to the generation of a denser and better organized connective tissue, especially in DR-EGF/2.0 group. These findings suggest a positive action of the rhEGF treatment in the improvement of diabetic healing. It must be also noted that the therapeutic effect of local rhEGF showed to be dose-dependent. At low doses (0.1 µg/mL), although promoting a better epithelialization, complete closure was not achieved in all animals, which could suggest a different sensibility to exogenous EGF depending on the individual. In addition, in this group neovascularization and collagenization were inferior when compared to those of DR-EGF/0.5 and DR-EGF/2.0 groups. However, the dose–response of rhEGF does not present either a simple linear pattern or a plateau of biological action, as high doses (8.0 µg/mL) elicited a disorganized growth of the reparative tissue at a dermal level.

EGF mechanism of action upon wound healing is complex, and many aspects remain unclear. Direct effects upon the cellular component of the tissue can be postulated, as well as indirect upon infiltrating inflammatory cells (17, 34). Considering the deep impact of the diabetic pathology upon the immune response (35), we aimed to investigate the effects of the rhEGF treatment upon wound T lymphocytes and macrophages, using the two most effective concentrations from the previous phase of our study. It could be clearly observed that macrophage infiltration in the repairing tissue was more intense in DRs than in healthy controls, and that it lasted longer. The abnormal behaviour observed in placebo-treated animals was reverted by the topical administration of rhEGF, which favoured the remission of the macrophage population. At the end of the study (day 21), rhEGF-treated animals showed a normalization of the macrophage presence, with values comparable to those of non-diabetic rats, while DR-PLCB group failed to resolve tissue inflammation. To further characterize the effects of rhEGF treatment, we also investigated the expression of several key cytokines in the reparative tissue by qRT-PCR. In diabetic animals, higher levels of mRNA were found for the proinflammatory cytokines TNF-α and IL-12p40, and for the immunoregulatory IL-10, indicating a greater activation than in healthy animals. Interestingly, TNF-α levels were downregulated over time, reaching normal values at day 21 both in placebo- and rhEGF-treated rats. Considering the importance of bacterial colonization and its products on macrophage activation and TNF-α production, as described by several authors (36, 37, 38), it can be suggested that the presence of infection is not the limiting factor of the healing. A similar phenomenon occurs in the expression of IL-10, which is considered to possess a reactive effect on macrophage activation, with a negative modulation of its persistence (39). However, the behaviour of IL-12p40 is different (40), and animals suffering from diabetes expressed sustained high levels of this cytokine, which was reversed to normal at the end of the study by treating with rhEGF. The presence of macrophages in the tissue was not accompanied by an elevated infiltration of CD4^+^ nor CD8^+^ T lymphocytes. Both types of lymphocytes were present only in small numbers, which was coherent with the observed low expression of IL-2. These data indicate that local administration of rhEGF has an immuno-modulatory effect, reducing the local inflammatory state of the healing tissue in diabetic animals. Although the mechanisms of action through which rhEGF exerts this action remain unknown, this activity seems to be related to an immuno-modulatory effect during wound healing.

In summary, the results of our work show that local administration of rhEGF possesses a novel therapeutic, immuno-modulatory effect upon diabetic cutaneous wound repair in an experimental model, thus being able to reduce the altered inflammatory response associated to diabetic healing.

## Declaration of interest

The authors declared no potential conflicts of interest with respect to the research, authorship and/or publication of the research reported.

## Funding

Funding was provided by Praxis Pharmaceutical, Madrid (B2017/BMD-3804, MITIC-CM) and National Institute of Health Carlos III (FIS-PI13/01413). rhEFG (Nepidermina) used in this research was kindly provided by Praxis Pharmaceutical S.A.

## Author contribution statement

G H N, J B, E G and M A M designed the study and experiments. G H N, A C and M A O carried out the RT-qPCR process, immunohistochemistry and statistical analyses. A C, M P and G G created the figures. G H N, A C, M A O, E G, J B and M A M edited the paper and commented on the interpretation of the results. J B, G G and M A M participated in study coordination and supervision. All authors read, discussed and approved the final manuscript.
